# Prevalence of Adverse Childhood Experiences and Long‐Term Associations With Mental Health Among Adults: A Nationwide Cross‐Sectional Study in China

**DOI:** 10.1002/mco2.70366

**Published:** 2025-09-01

**Authors:** Yi Guo, Yibo Wu, Siyuan Fan, Haibo Wang

**Affiliations:** ^1^ Peking University First Hospital Peking University Beijing China; ^2^ Clinical Research Institute Institute of Advanced Clinical Medicine Peking University Beijing China; ^3^ Ministry of Education Key Laboratory of Epidemiology of Major Diseases (Peking University) Beijing China; ^4^ Department of Nursing The Fourth Affiliated Hospital of School of Medicine and International School of Medicine International Institutes of Medicine Zhejiang University Hangzhou China; ^5^ School of Public Health Imperial College London London UK

**Keywords:** adverse childhood experience (ACE), anxiety, depression, suicidal ideation

## Abstract

This study aimed to estimate the prevalence of adverse childhood experiences (ACEs) and explore their associations with mental health among Chinese adults. This population‐based, cross‐sectional survey was conducted in China in 2023. Data on participants' ACEs, depressive symptoms, anxiety symptoms, suicidal ideation, and other information were collected among participants who were selected using multi‐stage stratified quota random sampling. Of 30,054 participants, 26.0% (7809/30,054) reported at least one ACE. The prevalence of major depression symptoms, moderate or severe anxiety symptoms, and suicidal ideation were 19.5%, 12.7%, and 21.7%, respectively. There was a dose‒response relationship between the cumulative number of ACEs and mental health among Chinese adults. Compared to those with no ACEs, the adjusted odds ratios (ORs) and 95% confidence interval for major depression symptoms were 1 ACE: 1.340 (1.226‒1.465), 2 ACEs: 1.769 (1.588‒1.971), 3 ACEs: 2.172 (1.909‒2.472), and ≥4 ACEs: 3.084 (2.712‒3.507). The ORs for anxiety symptoms of 1, 2, 3, and ≥4 ACEs were 1.262 (1.135‒1.403), 1.714 (1.513‒1.942), 2.119 (1.831‒2.452), and 2.890 (2.512‒3.325). The ORs for suicidal ideation were 1.056 (0.966‒1.154), 1.324 (1.188‒1.477), 1.470 (1.287‒1.679), and 3.134 (2.761‒3.557). Sexual abuse survivors were at great risk for mental health problems. Comprehensive measures are needed to support populations affected by ACEs.

## Graphical abstract

In this national cross‐sectional survey, our study showed that there was a dose‒response relationship between the cumulative number of ACEs and mental health among Chinese adults. Sexual abuse survivors were at great risk for mental health problems. Comprehensive measures are needed to support populations affected by ACEs.

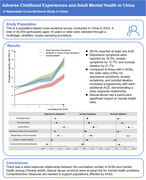

## 1 Introduction

Adverse childhood experiences (ACEs) are potentially traumatic events that occur in childhood and adolescence, such as experiencing abuse (physical, emotional, or sexual), witnessing violence, or growing up in a household with issues such as substance use, mental health problems, and parental incarceration [1]. ACEs exert profound and enduring effects on individuals' health and well‐being into adulthood, especially for mental problems including depression, anxiety, and suicide [2]. Existing research provides various potential neurobiological and psychosocial mechanisms through which ACEs affect mental health. Neurobiological mechanisms include dysfunction of the hypothalamic‒pituitary‒adrenal axis [3], alterations in brain structure and function [4], and epigenetic modifications [5]. Psychosocial mechanisms involve the development of maladaptive coping strategies [6], and the building of dysfunctional cognitions about the self and the world [7]. These factors collectively lead to long‐term impairments in emotion regulation and stress response. Research consistently identifies ACEs as a significant risk factor for the onset and exacerbation of depression in adulthood [8]. Furthermore, investigations worldwide have extensively examined the associations between ACEs and the manifestation of anxiety symptoms and suicidal behaviors [9, 10].

However, the majority of existing research was carried out in Western and high‐income countries. There were limited studies conducted in China [11], which focused primarily on specific population, such as middle‐aged and elderly people [12, 13], students [14], or patients with mental health disorders [15], lacking nationally representative population‐based survey of ACEs. Moreover, the available ACE‐related data in China has been incomplete and partial, gathered without the use of standardized assessment tools. A recent systematic review of child abuse research pointed out that Chinese studies mainly investigated single or few types of abuse, while surveys on all different types of abuse were scarce [16]. These studies tended to investigate specific ACE types, such as physical or emotional abuse. Comprehensive surveys, such as those utilizing the CHARLS database [12], also overlooked critical ACE categories such as sexual abuse. Understanding the prevalence of ACEs and its distribution patterns by sociodemographic characteristics, is indispensable to develop effective and evidence‐based strategies for addressing and preventing ACEs and eliminating disparities, so as to indirectly improve mental health in China [16].

Therefore, the current available data is insufficient to provide a comprehensive understanding of ACE prevalence and its association with the mental health of Chinese adults. Especially in a country such as China, with its unique social background and cultural diversity, the situation may differ from studies in western countries. For example, within a Confucian cultural context, victims of sexual abuse may experience heightened social stigma, intensifying psychological distress through mechanisms such as internalized shame [17] and barriers to seeking help [18]. In contrast, the psychological impact of corporal punishment may be less pronounced, as it is often regarded as a culturally normative parenting practice in many regions of China. The common saying, “Beating and scolding signify love,” reflects a perception among Chinese parents and children that such practices demonstrate care and involvement, potentially reducing associated feelings of shame or deviance [19, 20].

This study aims to address these research gaps by conducting a nationally representative cross‐sectional study among Chinese adults to estimate the prevalence of ACEs, mental health status (depression symptoms, anxiety symptoms, and suicidal ideation), and to explore the associations between ACEs and mental health status, so as to provide scientific evidence for the development of relevant intervention measures.

## 2 Results

### 2.1 Sociodemographic Characteristics of Participants

Among the 30,054 eligible participants included in the final analysis, average age was 43.3 ± 16.5 years. The majority of individuals (69.2%) had completed at least junior high school education. A total of 5.6% of participants reported experiencing a traumatic event in the past year. Using a cutoff score of 10 or higher, 5865 (19.5%) participants were screened positive for major depression symptoms, while 3826 (12.7%) participants were screened positive for moderate or severe anxiety symptoms. Additionally, 21.7% of participants reported suicidal ideation (Table 1).

**TABLE 1:** Sociodemographic and health characteristics of the sample by sex.

Characteristics	Sex		Total (*N* = 30,054)
	Male (*N* = 15,011)	Female (*N* = 15,043)	
Age	43.3 ± 16.6	43.3 ± 16.5	43.3 ± 16.5
Residence			
Rural	4650 (31.0%)	4669 (31.0%)	9319 (31.0%)
Urban	10,361 (69.0%)	10,374 (69.0%)	20,735 (69.0%)
Educational level			
≤Junior high	4245 (28.3%)	4998 (33.2%)	9243 (30.8%)
Senior high or specialty education	5473 (36.5%)	4724 (31.4%)	10,197 (33.9%)
≥Undergraduate	5293 (35.3%)	5321 (35.4%)	10,614 (35.3%)
Occupational status			
Employed	9197 (61.3%)	9148 (60.8%)	18,345 (61.0%)
Student	2566 (17.1%)	2115 (14.1%)	4681 (15.6%)
Retired	1905 (12.7%)	2323 (15.4%)	4228 (14.1%)
Unemployed	1343 (9.0%)	1457 (9.7%)	2800 (9.3%)
Average monthly household income per capita (¥)^a^			
≤3000	3960 (26.4%)	4821 (32.1%)	8781 (29.2%)
3001‒6000	6759 (45.0%)	6669 (44.3%)	13,428 (44.7%)
≥6001	4292 (28.6%)	3553 (23.6%)	7845 (26.1%)
Marital status			
Unmarried (single, divorced, or widowed)	5474 (36.5%)	5153 (34.3%)	10,627 (35.4%)
Married	9537 (63.5%)	9890 (65.7%)	19,427 (64.6%)
No. of siblings			
0 (only child)	3891 (25.9%)	3079 (20.5%)	6970 (23.2%)
≥1 (non‐only child)	11,120 (74.1%)	11,964 (79.5%)	23,084 (76.8%)
BMI			
<18.5 (underweight)	992 (6.6%)	2148 (14.3%)	3140 (10.5%)
18.5‒24.9 (normal)	10,515 (70.1%)	10,808 (71.9%)	21,323 (71.0%)
25.0‒29.9 (overweight)	3178 (21.2%)	1735 (11.5%)	4913 (16.4%)
≥30.0 (obese)	326 (2.2%)	352 (2.3%)	678 (2.3%)
Disease status^b^			
No chronic conditions	10,204 (68.0%)	10,532 (70.0%)	20,736 (69.0%)
One chronic condition	3101 (20.7%)	2881 (19.2%)	5982 (19.9%)
Two or more chronic conditions	1706 (11.4%)	1630 (10.8%)	3336 (11.1%)
Traumatic exposure			
No	14,192 (94.5%)	14,185 (94.3%)	28,377 (94.4%)
Yes	819 (5.5%)	858 (5.7%)	1677 (5.6%)
Depression symptoms			
Minimal (0‒4)	7375 (49.1%)	7244 (48.2%)	14,619 (48.6%)
Mild (5‒9)	4471 (29.8%)	5099 (33.9%)	9570 (31.8%)
Moderate (10‒14)	1778 (11.8%)	1628 (10.8%)	3406 (11.3%)
Moderately severe (15‒19)	983 (6.6%)	804 (5.3%)	1787 (6.0%)
Severe (≥20)	404 (2.7%)	268 (1.8%)	672 (2.2%)
Anxiety symptoms			
Minimal anxiety (0‒4)	8770 (58.4%)	8709 (57.9%)	17,479 (58.7%)
Mild anxiety (5‒9)	4119 (27.4%)	4630 (30.8%)	8749 (29.1%)
Moderate anxiety (10‒14)	1660 (11.1%)	1349 (9.0%)	3009 (10.0%)
Severe anxiety (≥15)	462 (3.1%)	355 (2.3%)	817 (2.7%)
Suicidal ideation			
No	11,453 (76.3%)	12,083 (80.3%)	23,536 (78.3%)
Yes	3558 (23.7%)	2960 (19.7%)	6518 (21.7%)

Abbreviation: BMI, body mass index.

^a^ Approximately less than US$410, US$410 to less than US$820, and US$820 or more.

^b^ Disease status include hypertension, diabetes, hyperlipidemia, coronary heart disease, stroke, respiratory system diseases, urinary system diseases, digestive system diseases, osteoporosis, arthritis, cancer, and other diseases.

### 2.2 Prevalence of Different Types of ACEs

Overall, 7809 (26.0%) participants experienced at least one type of ACEs before the age of 18 years, 3505 (11.7%) participants experienced one ACE, while 4304 (14.3%) participants experienced two or more ACEs (Table 2). The most common type of ACE was emotional abuse (16.6%), followed by physical abuse (10.6%), violent treatment of mother or stepmother in household (7.9%), and household substance abuse (7.2%). The participants with average monthly household income ≤3000 yuan had significant higher prevalence of ACEs compared to those with higher income (Table 3).

**TABLE 2:** Adverse childhood experience (ACE) score by sociodemographic characteristics.

Characteristic	Total no.	ACE score (the cumulative number of ACEs), % (95% CI)					*p*‐Value
		0	1	2	3	≥4	
Sex							
Male	15,011	73.7 (73.0‐74.4)	11.2 (10.7‐11.7)	6.8 (6.4‐7.2)	4.0 (3.7‐4.4)	4.3 (3.9‒4.6)	0.046
Female	15,043	74.4 (73.7‐75.1)	12.1 (11.6‐12.7)	6.0 (5.6‐6.4)	4.0 (3.6‐4.3)	3.5 (3.2‒3.8)	
Age (years)							
<30	7987	70.7 (69.7‐71.7)	14.2 (13.5‐15.0)	7.4 (6.8‐8.0)	4.2 (3.8‐4.7)	3.4 (3.1‒3.9)	<0.001
30‒39	5389	76.3 (75.2‐77.5)	11.4 (10.5‐12.2)	5.6 (5.0‐6.3)	2.8 (2.4‐3.3)	3.9 (3.4‒4.4)	
40‒49	6236	76.4 (75.3‐77.5)	11.1 (10.4‐12.0)	6.0 (5.4‐6.6)	3.4 (2.9‐3.8)	3.1 (2.7‒3.6)	
50‒59	5008	76.2 (75.0‐77.4)	10.5 (9.7‐11.4)	6.0 (5.4‐6.7)	4.2 (3.6‐4.7)	3.2 (2.7‒3.7)	
60‒69	2889	72.1 (70.4‐73.7)	10.2 (9.2‐11.4)	6.4 (5.5‐7.4)	6.1 (5.2‐7.0)	5.2 (4.4‒6.1)	
≥70	2545	71.7 (69.9‐73.5)	7.0 (6.0‒8.0)	7.0 (6.0‐8.0)	4.7 (3.9‐5.6)	7.3 (6.3‒8.3)	
Residence							
Rural	9319	74.1 (73.2‐75.0)	10.3 (9.7‐11.0)	6.7 (6.2‐7.2)	4.2 (3.8‐4.6)	4.6 (4.2‒5.1)	0.469
Urban	20,735	74.0 (73.4‐74.6)	12.3 (11.8‐12.7)	6.3 (6.0‐6.6)	3.9 (3.6‐4.2)	3.6 (3.3‒3.8)	
No. of siblings							
0	6970	75.2 (74.2‐76.2)	10.9 (10.2‐11.7)	6.1 (5.5‐6.6)	3.5 (3.1‐4.0)	4.3 (3.8‒4.8)	0.019
≥1	23,084	73.7 (73.1‐74.2)	11.9 (11.5‐12.3)	6.5 (6.2‐6.9)	4.2 (3.9‐4.4)	3.8 (3.5‒4.0)	
Educational level							
≤Junior high	9243	74.1 (73.1‐74.9)	10.3 (9.7‐10.9)	6.7 (6.2‐7.2)	5.0 (4.6‐5.5)	3.9 (3.6‒4.4)	0.268
Senior high or specialty education	10,197	74.5 (73.6‐75.3)	11.9 (11.3‐12.5)	6.3 (5.8‐6.8)	3.7 (3.3‐4.1)	3.7 (3.3‒4.1)	
≥Undergraduate	10,614	73.6 (72.7‐74.4)	12.6 (12.0‐13.3)	6.3 (5.9‐6.8)	3.4 (3.1‐3.8)	4.1 (3.7‒4.5)	
Occupational status							
Employed	18,315	75.7 (75.1‐76.3)	11.3 (10.8‐11.7)	6.0 (5.7‐6.4)	3.5 (3.2‐3.7)	3.6 (3.3‒3.9)	<0.001
Student	4681	67.9 (66.6‐69.3)	15.5 (14.5‐16.6)	8.4 (7.6‐9.2)	4.3 (3.7‐4.9)	3.9 (3.4‒4.5)	
Retired	4228	75.2 (73.8‐76.5)	9.6 (8.7‒10.5)	5.9 (5.2‐6.6)	5.2 (4.6‐5.9)	4.2 (3.6‒4.8)	
Unemployed	2800	71.5 (69.8‐73.2)	10.9 (9.8‐12.1)	6.8 (5.9‐7.8)	5.2 (4.4‐6.1)	5.6 (4.8‒6.5)	
Average monthly household income per capita (¥)							
≤3000	8781	69.1 (68.1‐70.1)	12.1 (11.4‐12.8)	7.7 (7.1‐8.3)	4.9 (4.5‐5.4)	6.2 (5.7‒6.8)	<0.001
3001‒6000	13,427	76.0 (75.2‐76.7)	11.2 (10.7‐11.8)	6.1 (5.7‐6.5)	3.7 (3.4‐4.1)	3.0 (2.7‒3.3)	
≥6001	7845	76.2 (75.2‐77.1)	11.9 (11.2‐12.7)	5.6 (5.1‐6.1)	3.5 (3.1‐3.9)	2.8 (2.5‒3.2)	
Marital status							
Unmarried (single) divorced or widowed)	19,427	77.1 (76.5‐77.7)	10.5 (10.1‐11.0)	5.8 (5.4‐6.1)	3.8 (3.5‐4.0)	2.9 (2.6‒3.1)	<0.001
Married	10,627	68.4 (67.5‐69.3)	13.7 (13.1‐14.4)	7.6 (7.1‐8.2)	4.4 (4.0‐4.8)	5.8 (5.4‒6.2)	
Total	30,054	74.0 (73.5‐74.5)	11.7 (11.3‐12.0)	6.4 (6.1‐6.7)	4.0 (3.8‐4.2)	3.9 (3.7‒4.1)	

**TABLE 3:** ACE prevalence by sociodemographic characteristics.

		Individual ACE, % (95% CI)						
Characteristic	Total no.	Emotional abuse	Physical abuse	Sexual abuse	Substance abuse in the household	Mental illness in the household	Mother treated violently	Incarcerated household member
Sex								
Male	15,011	16.7 (16.1‒17.3)	11.7 (11.2‒12.2)	5.3 (4.9‒5.7)	7.8 (7.4‒8.3)	4.9 (4.5‒5.2)	9.2 (8.7‒9.7)	2.7 (2.5‒3.0)
Female	15,043	16.6 (16.0‒17.2)	9.6 (9.1‒10.0)	5.7 (5.3‒6.1)	6.2 (5.9‒6.6)	4.8 (4.4‒5.1)	8.5 (8.1‒9.0)	2.0 (1.8‒2.3)
*p*‐Value		0.812	<0.001	0.134	<0.001	0.656	0.043	<0.001
Age (years)								
<30	7987	19.7 (18.8‒20.6)	10.3 (9.7‒11.0)	6.7 (6.2‒7.3)	6.2 (5.7‒6.7)	5.7 (5.2‒6.2)	7.7 (7.1‒8.3)	2.1 (1.8‒2.5)
30‒39	5389	15.0 (14.0‒15.9)	9.0 (8.2‒9.8)	6.0 (5.4‒6.7)	6.7 (6.1‒7.4)	4.3 (3.8‒4.9)	7.4 (6.8‒8.2)	2.4 (2.0‒2.8)
40‒49	6236	14.3 (13.4‒15.1)	10.0 (9.2‒10.7)	4.8 (4.2‒5.3)	6.2 (5.7‒6.9)	4.0 (3.6‒4.6)	7.3 (6.7‒8.0)	1.8 (1.5‒2.2)
50‒59	5008	14.9 (13.9‒15.9)	9.8 (9.0‒10.7)	4.4 (3.8‒5.0)	6.6 (5.9‒7.3)	4.0 (3.5‒4.6)	8.8 (8.0‒9.6)	2.1 (1.7‒2.5)
60‒69	2889	17.8 (16.4‒19.2)	13.8 (12.6‒15.1)	4.7 (4.0‒5.5)	9.1 (8.0‒10.2)	5.0 (4.2‒5.8)	12.9 (11.7‒14.2)	3.6 (3.0‒4.4)
≥70	2545	18.5 (17.0‒20.1)	14.5 (13.1‒15.9)	5.3 (4.5‒6.3)	10.8 (9.7‒12.1)	6.6 (5.7‒7.7)	14.8 (13.5‒16.3)	3.7 (3.0‒4.5)
*p*‐Value		<0.001	<0.001	<0.001	<0.001	<0.001	<0.001	<0.001
Residence								
Rural	9319	17.0 (16.2‒17.8)	11.2 (10.6‒11.9)	4.7 (4.2‒5.1)	8.5 (7.9‒9.1)	4.9 (4.5‒5.3)	10.5 (9.8‒11.1)	2.8 (2.5‒3.2)
Urban	20,735	16.5 (16.0‒17.0)	10.3 (9.9‒10.8)	5.9 (5.5‒6.2)	6.4 (6.1‒6.7)	4.8 (4.5‒5.1)	8.2 (7.8‒8.5)	2.2 (2.0‒2.4)
*p*‐Value		0.280	0.018	<0.001	<0.001	0.74	<0.001	0.001
No. of siblings								
0	6970	16.3 (15.4‒17.2)	10.1 (9.4‒10.8)	6.4 (5.8‒7.0)	6.6 (6.0‒7.2)	5.5 (5.0‒6.1)	8.3 (7.7‒9.0)	2.5 (2.2‒2.9)
≥1	23,084	16.7 (16.3‒17.2)	10.8 (10.4‒11.2)	5.2 (4.9‒5.5)	7.2 (6.8‒7.5)	4.6 (4.3‒4.9)	9.0 (8.7‒9.4)	2.3 (2.1‒2.5)
*p*‐Value		0.357	0.091	<0.001	0.106	0.001	0.06	0.361
Educational level								
≤Junior high	9243	16.6 (15.9‒17.4)	12.0 (11.3‒12.7)	3.4 (3.0‒3.8)	8.1 (7.6‒8.7)	4.1 (3.7‒4.6)	11.0 (10.4‒11.7)	2.4 (2.1‒2.7)
Senior high or specialty education	10,197	16.4 (15.7‒17.1)	10.0 (9.4‒10.6)	5.5 (5.1‒6.0)	7.2 (6.7‒7.7)	4.6 (4.2‒5.0)	7.9 (7.4‒8.5)	2.3 (2.0‒2.6)
≥Undergraduate	10,614	16.9 (16.1‒17.6)	10.1 (9.5‒10.6)	7.3 (6.8‒7.8)	6.0 (5.5‒6.4)	5.7 (5.2‒6.1)	7.9 (7.4‒8.4)	2.4 (2.1‒2.7)
*p*‐Value		0.663	<0.001	<0.001	<0.001	<0.001	<0.001	0.896
Occupational status								
Employed	18,315	15.2 (14.6‒15.7)	9.8 (9.4‒10.2)	5.4 (5.1‒5.7)	6.6 (6.3‒7.0)	4.3 (4.0‒4.6)	7.9 (7.6‒8.3)	2.1 (1.9‒2.4)
Student	4681	21.6 (20.5‒22.8)	11.3 (10.4‒12.2)	7.2 (6.5‒8.0)	6.3 (5.6‒7.1)	6.7 (6.0‒7.4)	8.5 (7.7‒9.3)	2.4 (2.0‒2.9)
Retired	4228	15.6 (14.5‒16.7)	12.2 (11.2‒13.2)	3.8 (3.3‒4.5)	7.9 (7.1‒8.7)	4.6 (4.0‒5.2)	11 (10.1‒12.0)	2.8 (2.3‒3.3)
Unemployed	2800	19.5 (18.0‒21.0)	12.4 (11.2‒13.7)	5.8 (4.9‒6.7)	9.7 (8.6‒10.8)	5.7 (4.9‒6.6)	12.3 (11.1‒13.6)	3.4 (2.7‒4.1)
*p*‐Value		<0.001	<0.001	<0.001	<0.001	<0.001	<0.001	<0.001
Average monthly household income per capita (¥)								
≤3000	8781	20.3 (19.5‒21.2)	13.6 (12.9‒14.4)	7.1 (6.5‒7.6)	9.8 (9.1‒10.4)	6.8 (6.3‒7.4)	12.5 (11.8‒13.2)	3.6 (3.2‒4.0)
3001‒6000	13,427	15.4 (14.8‒16.1)	9.6 (9.1‒10.1)	4.4 (4.0‒4.7)	6.1 (5.7‒6.6)	3.9 (3.6‒4.2)	7.8 (7.3‒8.2)	2.0 (1.7‒2.2)
≥6001	7845	14.6 (13.8‒15.4)	8.9 (8.3‒9.6)	5.6 (5.1‒6.2)	5.5 (5.0‒6.0)	4.2 (3.8‒4.7)	6.7 (6.2‒7.3)	1.7 (1.4‒2.0)
*p*‐Value		<0.001	<0.001	<0.001	<0.001	<0.001	<0.001	<0.001
Marital status								
Unmarried (single, divorced, or widowed)	19,427	14.3 (13.8‒14.8)	9.6 (9.2‒10.0)	3.9 (3.6‒4.2)	6.1 (5.8‒6.5)	3.5 (3.2‒3.7)	7.9 (7.6‒8.3)	1.8 (1.6‒2.0)
Married	10,627	20.9 (20.2‒21.7)	12.5 (11.9‒13.1)	8.4 (7.9‒9.0)	8.7 (8.2‒9.2)	7.3 (6.8‒7.8)	10.6 (10.0‒11.2)	3.5 (3.2‒3.9)
*p*‐Value		<0.001	<0.001	<0.001	<0.001	<0.001	<0.001	<0.001
Total	30,054	16.6 (16.2‒17.1)	10.6 (10.3‒11.0)	5.5 (5.2‒5.8)	7.0 (6.7‒7.3)	4.8 (4.6‒5.1)	8.9 (8.5‒9.2)	2.4 (2.2‒2.6)

### 2.3 Dose‒Response Relationships between ACEs and Mental Health Outcomes

**FIGURE 1:**
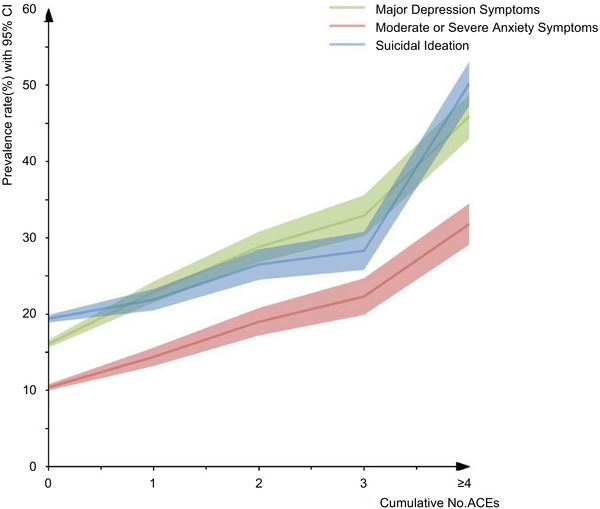
Relationship between ACE score and mental health status (screened positive for major depression symptoms, moderate or severe anxiety symptoms, and suicidal ideation).

We observed a dose‒response relationship between ACE categories and the prevalence rate of major depression symptoms, moderate or severe anxiety symptoms, and suicidal ideation (Figure 1). The adjusted odds ratios (ORs) for depression symptom, anxiety symptom, and suicidal ideation increased gradually with the total number of ACEs. Compared with those participants who reported no ACEs, the adjusted ORs (95% confidence interval [CI]) of 1, 2, 3, and ≥4 ACEs for depression symptom were 1.340 (1.226‒1.465), 1.769 (1.588‒1.971), 2.172 (1.909‒2.472), and 3.084 (2.712‒3.507), respectively; those for the level of anxiety symptom were 1.262 (1.135‒1.403), 1.714 (1.513‒1.942), 2.119 (1.831‒2.452), and 2.890 (2.512‒3.325), respectively. The adjusted OR for suicidal ideation did not reach statistical significance for participants reporting one ACE, but for those reporting 2, 3, and ≥4 ACEs, the ORs for suicidal ideation were 1.324 (1.188‒1.477), 1.470 (1.287‒1.679), and 3.134 (2.761‒3.557), respectively (Table 4). The subgroup analyses also showed consistent trends in the dose‒response relationship across age groups in which most of the statistical analyses revealed significant associations. However, the dose‒response associations between ACE categories and mental health status decreased gradually with age, except for the participants aged ≥70 years among whom the association rebounded (Table 5).

**TABLE 4:** Adjusted association between ACE categories and mental health outcomes by logistic regression model.

Characteristic	Depression symptoms		Anxiety symptoms		Suicidal ideation	
	Adjusted OR (95% CI)	*p*‐Value	Adjusted OR (95% CI)	*p*‐Value	Adjusted OR (95% CI)	*p*‐Value
ACE category (ref: 0)	‒	‒	‒	‒	‒	‒
1	1.340 (1.226‒1.465)	<0.001	1.262 (1.135‒1.403)	<0.001	1.056 (0.966‒1.154)	0.232
2	1.769 (1.588‒1.971)	<0.001	1.714 (1.513‒1.942)	<0.001	1.324 (1.188‒1.477)	<0.001
3	2.172 (1.909‒2.472)	<0.001	2.119 (1.831‒2.452)	<0.001	1.470 (1.287‒1.679)	<0.001
≥4	3.084 (2.712‒3.507)	<0.001	2.890 (2.512‒3.325)	<0.001	3.134 (2.761‒3.557)	<0.001
Age	0.991 (0.988‒0.994)	<0.001	0.990 (0.986‒0.994)	<0.001	0.997 (0.994‒0.999)	0.022
Sex (ref: male)	‒	‒	‒	‒	‒	‒
Female	0.826 (0.778‒0.878)	<0.001	0.792 (0.737‒0.850)	<0.001	0.803 (0.758‒0.850)	<0.001
Residence (ref: rural)	‒	‒	‒	‒	‒	‒
Urban	0.877 (0.819‒0.941)	<0.001	0.877 (0.808‒0.951)	0.002	0.848 (0.794‒0.906)	<0.001
Educational level (ref: ≤junior high)	‒	‒	‒	‒	‒	‒
Senior high or specialty education	1.322 (1.213‒1.441)	<0.001	1.239 (1.119‒1.372)	<0.001	1.412 (1.301‒1.533)	<0.001
≥Undergraduate	1.299 (1.180‒1.431)	<0.001	1.199 (1.070‒1.343)	0.002	1.441 (1.315‒1.580)	<0.001
Occupational status (ref: employed)	‒	‒	‒	‒	‒	‒
Student	0.789 (0.715‒0.871)	<0.001	0.758 (0.676‒0.850)	<0.001	0.722 (0.655‒0.795)	<0.001
Retired	1.132 (1.011‒1.267)	0.032	1.175 (1.029‒1.341)	0.017	1.007 (0.905‒1.122)	0.893
Unemployed	1.260 (1.130‒1.404)	<0.001	1.243 (1.094‒1.411)	0.001	1.226 (1.105‒1.360)	<0.001
Average monthly household income per capita (¥) (ref: ≤3000)	‒	‒	‒	‒	‒	‒
3001‒6000	0.872 (0.811‒0.937)	<0.001	0.892 (0.819‒0.972)	0.009	0.880 (0.822‒0.944)	<0.001
≥6001	0.915 (0.841‒0.995)	0.039	1.019 (0.923‒1.124)	0.714	0.915 (0.844‒0.992)	0.032
Marital status (ref: married)	‒	‒	‒	‒	‒	‒
Unmarried	1.373 (1.272‒1.482)	<0.001	1.350 (1.235‒1.476)	<0.001	1.319 (1.226‒1.419)	<0.001
No. of siblings (ref: 0)	‒	‒	‒	‒	‒	‒
≥1	0.977 (0.909‒1.050)	0.526	0.898 (0.826‒0.976)	0.011	0.875 (0.817‒0.936)	<0.001
BMI (ref: <18.5, underweight)	‒	‒	‒	‒	‒	‒
18.5 ≤ BMI < 25.0 (normal)	0.889 (0.808‒0.977)	0.015	0.936 (0.837‒1.048)	0.254	0.857 (0.782‒0.939)	0.001
25.0 ≤ BMI < 30.0 (overweight)	0.950 (0.845‒1.068)	0.389	0.964 (0.840‒1.107)	0.606	0.918 (0.821‒1.027)	0.133
BMI ≥ 30.0 (obese)	1.092 (0.893‒1.334)	0.391	1.094 (0.866‒1.383)	0.45	1.015 (0.835‒1.234)	0.883
Disease status (ref: not afflicted)	‒	‒	‒	‒	‒	‒
Afflicted with one condition	1.090 (1.004‒1.184)	0.041	1.136 (1.031‒1.251)	0.01	1.036 (0.958‒1.121)	0.377
Afflicted with two or more conditions	1.398 (1.262‒1.548)	<0.001	1.327 (1.177‒1.496)	<0.001	1.253 (1.135‒1.382)	<0.001
Traumatic exposure (ref: no)	‒	‒	‒	‒	‒	‒
Yes	1.577 (1.480‒1.680)	<0.001	1.533 (1.422‒1.652)	<0.001	1.381 (1.300‒1.467)	<0.001

**TABLE 5:** Age‐based subgroup analysis for adjusted association between ACE and mental health by logistic regression model.

Mental health status	Age group (years)	ACE category (ref: 0)			
		1	2	3	≥4
Depression symptoms	<30	1.620 (1.394‒1.883)	2.266 (1.880‒2.731)	3.071 (2.437‒3.869)	4.149 (3.201‒5.378)
	30‒39	1.275 (1.038‒1.566)	2.003 (1.551‒2.586)	2.353 (1.667‒3.323)	2.048 (1.501‒2.793)
	40‒49	1.267 (1.028‒1.561)	1.597 (1.237‒2.063)	2.598 (1.912‒3.532)	3.355 (2.451‒4.592)
	50‒59	1.087 (0.844‒1.399)	1.297 (0.952‒1.769)	1.795 (1.282‒2.513)	3.254 (2.299‒4.606)
	60‒69	1.085 (0.772‒1.524)	1.383 (0.936‒2.045)	1.426 (0.959‒2.119)	1.810 (1.205‒2.719)
	≥70	1.452 (1.030‒2.047)	1.568 (1.072‒2.294)	1.225 (0.765‒1.963)	1.821 (1.240‒2.675)
Anxiety symptoms	<30	1.475 (1.232‒1.766)	2.220 (1.796‒2.744)	2.878 (2.231‒3.713)	3.683 (2.802‒4.842)
	30‒39	1.210 (0.952‒1.537)	1.880 (1.412‒2.504)	2.199 (1.509‒3.205)	2.431 (1.752‒3.372)
	40‒49	1.321 (1.031‒1.692)	1.887 (1.417‒2.514)	2.536 (1.787‒3.598)	2.395 (1.675‒3.424)
	50‒59	0.926 (0.679‒1.263)	1.074 (0.736‒1.568)	1.724 (1.174‒2.532)	3.092 (2.134‒4.480)
	60‒69	1.119 (0.746‒1.678)	1.392 (0.877‒2.211)	1.260 (0.775‒2.047)	1.940 (1.223‒3.076)
	≥70	1.429 (0.941‒2.169)	1.051 (0.628‒1.758)	1.522 (0.889‒2.606)	1.496 (0.941‒2.378)
Suicidal ideation	<30	1.333 (1.143‒1.555)	1.798 (1.485‒2.178)	2.442 (1.931‒3.088)	4.456 (3.436‒5.779)
	30‒39	0.983 (0.802‒1.205)	1.384 (1.070‒1.791)	1.604 (1.133‒2.272)	2.937 (2.163‒3.988)
	40‒49	0.959 (0.780‒1.179)	1.266 (0.984‒1.628)	1.665 (1.214‒2.283)	2.285 (1.667‒3.131)
	50‒59	0.964 (0.756‒1.228)	0.955 (0.699‒1.306)	1.048 (0.732‒1.500)	2.269 (1.604‒3.209)
	60‒69	0.685 (0.478‒0.983)	0.912 (0.607‒1.371)	0.859 (0.560‒1.318)	2.026 (1.364‒3.009)
	≥70	1.213 (0.868‒1.696)	1.257 (0.859‒1.839)	0.804 (0.489‒1.321)	2.295 (1.590‒3.313)

*Note*: Adjusted for sex, educational level, occupational status, income per capita, marital status, no. of siblings, BMI, and disease status, and traumatic exposure.

### 2.4 Sociodemographic and Clinical Correlates of Mental Health Outcomes

Additionally, the regression models revealed that having a higher level of education, being unmarried, having diseases, and with traumatic exposure were associated with more severe depression symptoms, anxiety symptoms, and suicidal ideation (Table 4). On the other hand, older age, being a student, and having a higher average monthly household income were associated with lower risk of depression symptoms, anxiety symptoms, and suicidal ideation. Furthermore, non‐only children tend to have milder anxiety symptoms and suicidal ideation, while being female and residing in urban areas are negatively correlated with suicidal ideation.

### 2.5 Impact of Individual ACEs on Mental Health Outcomes

**FIGURE 2:**
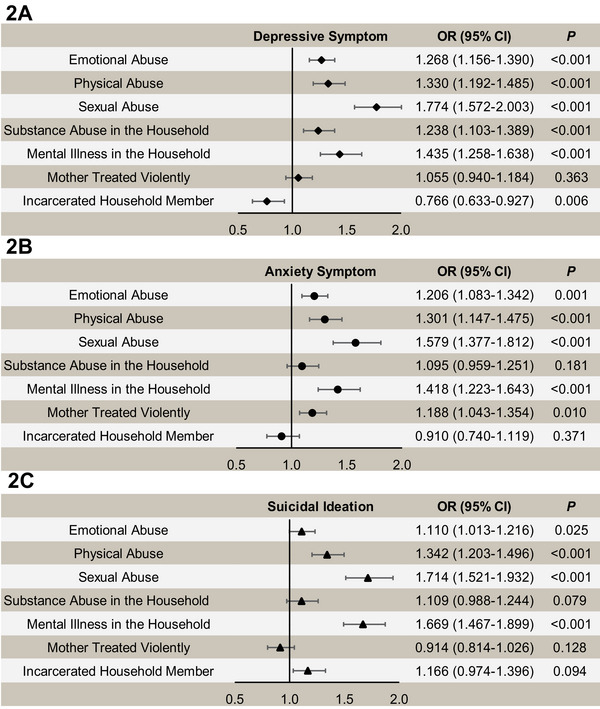
Adjusted ORs (95% CIs) for the association between individual ACEs and major depression symptoms (A), moderate or severe anxiety symptoms (B), and suicidal ideation (C) by logistic regression model. *Note*: Adjusted for age, sex, residence, educational level, occupational status, average monthly household income per capita, marital status, number of siblings, BMI, disease status, and traumatic exposure.

The study findings also revealed significant associations between individual ACEs and depression symptoms, anxiety symptoms, and suicidal ideation (Figure 2). ACEs closely linked to depression symptoms included sexual abuse (OR 1.774; 95% CI 1.572‒2.003), mental illness in the household (OR 1.435; 95% CI 1.258‒1.638), and emotional abuse (OR 1.268; 95% CI 1.156‒1.390). ACEs closely associated with anxiety symptoms included sexual abuse (OR 1.579; 95% CI 1.377‒1.812), mental illness in the household (OR 1.418; 95% CI 1.223‒1.643), and physical abuse (OR 1.301; 95% CI 1.147‒1.475). ACEs closely linked to suicidal ideation included sexual abuse (OR 1.714; 95% CI 1.521‒1.932), mental illness in the household (OR 1.669; 95% CI 1.467‒1.899), and physical abuse (OR 1.342; 95% CI 1.203‒1.496). Sexual abuse demonstrated the highest ORs among individual ACEs in the regression analyses, with effect sizes significantly exceeding most other predictors (*p* < 0.05), and retaining higher point estimates in the most instances where statistical significance was observed (Table 6).

**TABLE 6:** Wald test results comparing effect sizes of sexual abuse and other factors.

Outcome	Predictor factor	Wald *χ* ^2^	*p*‐Value
Depression symptoms	Emotional abuse	17.70	<0.001
	Physical abuse	11.53	<0.001
	Substance abuse in the household	16.17	<0.001
	Mental illness in the household	4.62	0.032
	Mother treated violently	33.99	<0.001
	Incarcerated household member	45.78	<0.001
Anxiety symptoms	Emotional abuse	8.69	0.003
	Physical abuse	4.03	0.045
	Substance abuse in the household	12.68	<0.001
	Mental illness in the household	0.94	0.333
	Mother treated violently	7.92	0.005
	Incarcerated household member	16.27	<0.001
Suicidal ideation	Emotional abuse	30.04	<0.001
	Physical abuse	8.49	0.004
	Substance abuse in the household	23.98	<0.001
	Mental illness in the household	0.08	0.783
	Mother treated violently	49.79	<0.001
	Incarcerated household member	10.62	0.001

## 3 Discussion

In this nationally representative cross‐sectional study of adults across China, we have investigated the prevalence of ACEs, depression symptoms, anxiety symptoms, and suicidal ideation, and demonstrated a dose‒response relationship between the cumulative number of ACEs and the levels of depression symptoms, anxiety symptoms, and suicidal ideation. Furthermore, we also found significant associations between individual ACEs and mental health.

As we know, this was the first nationwide representative survey of ACEs prevalence among the general adult population in China. The results showed that 11.7% participants had experienced one ACE and 14.3% had experienced ≥2 ACEs. The study also found that among various types of ACEs, emotional abuse had the highest prevalence of 16.6%, which was similar to the most recent meta‐analysis of ACE burden in China (19.6%, 95% CI 15.4–23.7), but lower than that in North America (34.4%, 95% CI 33.8‒35.0). The variations for ACE prevalence may be explained in part because of the different survey tool, in addition to the different sampling methods to select the participants. The prevalence of sexual abuse was 5.5%, similar to findings in high‐income countries [21]. However, the prevalence among Chinese women was much lower than the typical 10%‒30% reported for western women, while the prevalence among Chinese men ranged from 3% to 10% comparable to western counterparts [22]. This difference may reflect cultural traditions in China that prioritize protecting children, especially girls, from childhood sexual contact. However, underreporting by females may also play a role due to Chinese traditional culture that keeps such experiences secret to preserve their own and their family's honor [23].

This study also found a prevalence of 19.5% for major depression symptoms and 12.7% for moderate or severe anxiety symptoms. The prevalence of major depression symptoms was slightly higher than the 15.0% prevalence reported in previous meta‐analyses of Asian populations [24], but similar to the 18.0% prevalence reported in the previous Chinese Dongfeng‐Tongji cohort study [25]. Additionally, this study first reported a relatively high prevalence of suicidal ideation (21.7%) among a nationally representative sample of adults. Although some surveys on suicidal ideation among the Chinese population have been conducted previously, most were targeted at specific groups such as adolescents or college students, with the proportions ranging from 10.7% to 32.0% [26, 27], and our study extends these findings to the broader adult population. These findings highlight the seriousness and pervasiveness of mental health issues among Chinese adults. It is worth noting that the prevalence of severe depressive symptoms is slightly higher than those in some previous studies [24, 25], partly due to the impact of coronavirus disease 2019 (COVID‐19) on the mental health of Chinese adults in recent years. Additionally, the prevalence of moderate to severe anxiety symptoms should not be overlooked, as their impact on individual quality of life and social functioning is equally significant. This study also reveals, for the first time, the high prevalence of suicidal ideation among Chinese adults. It was worth noting that we used item 9 of the patient health questionnaire‐9 (PHQ‐9) to assess suicidal ideation, which captures a broad spectrum of ideation, including passive or fleeting thoughts of death, potentially contributing to higher prevalence estimates. However, it has been widely used in population‐based surveys as a reliable screening tool for its sensitivity in identifying individuals at risk. Unfortunately, suicidal ideation screening is not common in primary healthcare settings across China. To address this critical gap in mental healthcare, we strongly recommend implementing standardized suicide risk assessment in primary care systems, coupled with a tiered referral system for specialized psychiatric services. Such improvements would facilitate earlier identification of high‐risk individuals and timely delivery of psychological support. The prevalence of major depressive disorder or generalized anxiety disorder according to clinical diagnostic criteria should be further investigated in the future as the scores of PHQ‐9 and generalized anxiety symptoms‐7 (GAD‐7) only reflect the current presence of depressive or anxiety symptoms, although widely used in the screening.

Our research findings revealed a dose‒response relationship between ACE categories and the risk of depression symptoms, anxiety symptoms, and suicidal ideation. These findings were consistent with those conducted in other regions [28, 29]. Especially, adults who have experienced four or more ACEs have threefold higher likelihood of suicidal ideation compared to those without ACEs, highlighting the need for targeted and urgent measures to prevent suicidal ideation among individuals who have experienced multiple ACEs. The dose‒response relationship was further confirmed across different age groups by subgroup analysis; however, the association coefficients decreased gradually with age, which was consistent with previous studies of ACEs [30]. Both recall bias and the natural tapering of long‐term effect of ACEs may be important underlying mechanisms. And we speculated the mild bounce of association among elder participants (≥70 years) was due to more severe ACEs with impressive memory they experienced in an extraordinary period (World War II and civil war). Notably, more ACEs were reported by participants ≥70 years compared with participants aged 60‒69 years. As an exceptional finding, there was a small but not statistically significant increase in risk of suicidal ideation among participants with only one ACE. However, some studies suggested that accumulating just one ACE may significantly increase the risk of suicidal tendencies among young people [31]. Additionally, studies in the general population in the United States have also concluded that even one ACE can significantly impact suicidal ideation [32]. These differences may be related to factors such as the study population, cultural background, and methodological design. In the future, further research is needed to clarify the conclusion when interpreting the association between one ACE and suicidal ideation.

When exploring the relationship between different types of ACEs and mental health, it was found that sexual abuse emerged as the strongest predictor for depression symptoms, anxiety symptoms, and suicidal ideation among individual ACEs. The significant and robust associations of sexual abuse with mental illness highlighted the importance of this dimension, which was previously overlooked in many ACE studies in China [12]. Sexual abuse exhibits the most significant associations within individual ACEs, potentially for multiple reasons. On one hand, survivors of sexual abuse often encounter interpersonal disruptions compared to those who have not experienced similar events, ultimately leading to reduced social support, making it more challenging for them to cope with the negative consequences of sexual abuse [33]. On the other hand, this issue is closely related to the cultural background of China [32]. Most regions maintain a traditional Confucian cultural environment characterized by conservative attitudes toward sexuality. Within this cultural context, survivors of sexual abuse often face stigmatization and discrimination [34], further exacerbating their psychological trauma. Considering the Confucian cultural heritage in China, it would be helpful to strengthen public education to reduce social stigma, create a safe disclosure environment for victims, especially women, and implement culturally sensitive sex education programs in both community settings and schools.

Furthermore, research findings indicate that, among various types of ACE, besides the strong association with sexual abuse, the presence of mental illness among family members also plays a significant role. Individuals with a family history of mental illness are more likely to experience symptoms of depression, anxiety, and suicidal ideation in adulthood, consistent with previous studies [35, 36]. However, there is a striking lack of global evidence on parenting interventions for the offspring of individuals with severe mental illnesses [37]. Limited studies suggest that interventions such as offering specialized psychological counseling services tailored to this group may help mitigate their psychological risks [38]. Future studies should prioritize longitudinal research to establish stronger causal evidence for developing effective interventions.

### 3.1 Limitations

This study was subject to several limitations. First, the cross‐sectional design inherently limits our ability to establish causality. However, the limitation is partially mitigated since ACEs occurred before the age of 18 preceding the adult outcomes. Second, the retrospective collection of self‐reported ACEs might introduce some degree of recall bias, but the impact is likely modest as a well‐validated ACE questionnaire was used in the study and the observed ACE prevalence rates showed remarkable consistency with other studies. Third, the study did not collect all types of ACEs including emotional neglect or physical neglect, although standardized assessment tools were employed. Fourth, the potential impact of COVID‐19 on mental health cannot be neglected. However, the association of ACEs with mental health was less affected due to non‐differential effect of COVID‐19 among the general population. Moreover, our survey tool may underestimate some ACEs. As corporal punishment is still prevalent in China, the participants may underestimate the extent of their experiences about physical abuse, as they may regard such behavior as customary [39]. Additionally, while we adjusted for multiple important confounders in examining the associations between ACEs and mental health outcomes, our study did not account for certain potential confounding variables, such as parental educational level and childhood family economic status. The absence of these variables may have led to residual confounding, potentially diminishing the associations between ACE exposure and health outcomes. Furthermore, there may be interactions between different ACEs, and future research can explore this further. Addressing these limitations in future research could enhance the robustness of findings and provide a more comprehensive understanding of the relationship between ACEs and health outcomes.

## 4 Conclusion

ACEs were common, with 26.0% of participants experienced at least one type of ACE in this nationally representative population‐based study. ACEs were not equally distributed, with different patterns by sociodemographical characteristics. There was a relatively high prevalence of suicidal ideation (21.7%) in the population. The study revealed a dose‒response relationship between the cumulative number of ACEs and depression symptoms, anxiety symptoms, and suicidal ideation among Chinese adults. Among individual ACEs, sexual abuse showed the strongest association with the aforementioned mental illness. Comprehensive measures should be taken to identify populations more affected by ACES and to reduce the risk exposed to ACEs.

## 5 Materials and Methods

### 5.1 Study Setting and Population

This was a population‐based cross‐sectional survey (Psychology and Behavior Investigation of Chinese Residents, PBICR) conducted in China from June 20 to August 31, 2023. It implemented a multistage, stratified, cluster‐sampling procedure. In stage 1, we randomly selected 160 cities/districts proportional to population size (cities from provinces and autonomous regions, districts from municipalities, and special administrative regions). In stage 2, we randomly selected three urban communities and two rural villages from each city/district. In stage 3, we recruited and screened potential eligible individuals (60 participants in each community/village) on the street. In stage 4, to resolve high proportion of younger people because of street‐based sample, we adopted post‐survey quota sampling which was set on age (5 years interval) and sex that approximated China's Seventh National Census [40]. A total of 30,054 participants from 800 different communities/villages were ultimately included in the final analyses. This study followed the Strengthening the Reporting of Observational Studies in Epidemiology reporting guideline.

The survey included participants who met the following inclusion criteria: (1) aged 18 years or older, (2) Chinese nationality, (3) permanent resident of China (with no more than 1 month of time spent outside the country per year), (4) willing to participate in the study and signed informed consent form, (5) able to complete the online questionnaire independently or with the assistance of the investigator, and (6) understanding the meaning of each item on the questionnaire. The exclusion criteria included: (1) with a history of schizophrenia, obsessive‐compulsive disorder, or bipolar disorder, or currently in the acute phase of these conditions, (2) having cognitive impairment (e.g., Alzheimer's disease) that prevents full comprehension of the questionnaire, and (3) participation in other similar surveys or previous involvement in the PBICR survey.

### 5.2 Data Collection

The data were gathered through online electronic questionnaires by using the *Wenjuanxing* application (a professional platform) and distributed via the social media platform WeChat. For participants unable to independently complete electronic questionnaires using smartphones (e.g., illiteracy, with visual or physical disabilities), investigators conducted face‐to‐face interviews and completed the questionnaires on their behalf in a private and comfortable environment. The survey was conducted anonymously to ensure privacy, and no identifiable information, such as name, address, or phone number, was collected. The questionnaire was designed with neutral, non‐leading questions to avoid eliciting socially desirable responses. All investigators received training that covered how to communicate using simple, clear, and non‐judgmental language, and identify and manage participants' emotional responses to reduce reporting bias on sensitive issues. All data were kept secretly with access limited to authorized research personnel.

The questionnaire included demographical characteristics, body mass index (BMI), self‐reported disease status (if known), traumatic exposure in the past year, ACEs, PHQ‐9, and GAD‐7.

The disease status included hypertension, diabetes, hyperlipidemia, coronary heart disease, stroke, respiratory system diseases, urinary system diseases, digestive system diseases, osteoporosis, arthritis, cancer, and other diseases. Traumatic exposure investigated injuries and negative life experiences within the past year. Injuries included motor vehicle accidents, falls, blunt instrument injuries, burns/scalds, suffocation, drowning, poisoning, sexual assault, etc. Negative life experiences covered events such as the death of family members, unemployment, legal disputes, financial difficulties, unexpected threats, accidents, natural disasters, property loss, serious illnesses, etc.

We used the ACE questionnaire from previous study [36], including three categories of childhood abuse (emotional abuse, physical abuse, and sexual abuse) and four categories of household dysfunction (substance abuse in the household, mental illness in the household, mother treated violently, and incarcerated household member). Respondents were defined as experiencing the ACE if they responded “yes” to any one of the questions in that category. We evaluated the content validity of the questionnaire's translation through expert consultation. This process included multiple rounds of discussion and revision to ensure that each item of the translated questionnaire accurately reflected the intent of the original questionnaire. The Cronbach's alpha of the ACE questionnaire in this study was 0.862.

We assessed the depression symptoms using PHQ‐9. Total scores of 5, 10, 15, and 20 were defined as cut points for mild, moderate, moderately severe, and severe depression symptoms, respectively [41]. We referred to a score of 10 or above as major depression symptoms (including moderately severe and severe depression symptoms). In addition, Any positive (≥1) response to the ninth item of PHQ‐9, which evaluated the thoughts of death or self‐injury within the past two weeks, was considered as having suicidal ideation [42]. The Chinese version of the PHQ‐9 has been validated in primary care settings, with internal consistency (Cronbach's alpha) being 0.89. The optimal cutoff score of 10 yields a sensitivity of 0.87 and a specificity of 0.81 [43].

GAD‐7 was used to evaluate anxiety symptoms. Total scores of 5, 10 and 15 were defined as cut points for mild, moderate and severe anxiety symptoms, respectively [44]. We recorded responses dichotomously based on a cutoff score of 10 to determine moderate or severe anxiety symptoms. The Chinese version of the GAD‐7 is also reliable and valid, with Cronbach's alpha being 0.937. A cutoff score of 10 can detect GAD with a sensitivity of 86.2% and a specificity of 95.5% [45].

### 5.3 Statistical Analysis

Consistent with prior studies [46], the cumulative number of ACEs was calculated and grouped into these categories: 0, 1, 2, 3, and ≥4 ACEs. We calculated the prevalence of ACEs (both each type and ACE category) and 95% CI by sociodemographic characteristics. The prevalence of major depression symptoms, moderate or severe anxiety symptoms, and suicidal ideation, and 95% CI were also calculated by ACE categories. Chi‐square test was used to compare the differences in the prevalence of individual ACE, and Kruskal‒Wallis or Wilcoxon rank sum tests were used to analyze the difference in ACE categories.

Binary logistic regression model was performed to estimate the independent association of individual ACEs, ACE categories with depression symptoms, anxiety symptoms, and suicidal ideation, adjusting for the following covariates (potential confounding factors because of the established associations with mental health): age, sex, residence, educational level, occupational status, average monthly household income per capita, marital status, number of siblings, BMI, disease status, and traumatic exposure. The Wald test was used to compare the effect sizes of various predictors. Subgroup analysis based on age groups was also performed.

All statistical analyses were performed using STATA 15.0 (Stata Corp., College Station. TX, USA). In all analyses, a two‐tailed value of *p* < 0.05 was considered statistically significant.

## Author Contributions

Y.G. contributed to data analysis, methodology development, and writing. Y.W. was involved in project administration and provided resources for the study. S.F. conducted investigation and curated the data for the study. H.W. secured funding for the research, conducted review and editing of the manuscript, validated the findings, and provided supervision. All the authors have read and approved the final manuscript.

## Ethics Statement

The research protocol was approved by the Shandong Provincial Hospital Ethics Review Board (SWYX: NO.2023‐198) and was registered in the Chinese Clinical Trial Registry (registration number ChiCTR2300072573). Written informed consent was obtained from all participants prior to their participation in the survey.

## Conflicts of Interest

The authors declare no conflicts of interest.

## Data Availability

The datasets generated during the current study are available from the corresponding author upon reasonable request.

## References

[1] National Center for Injury Prevention and Control of United States , Preventing Adverse Childhood Experiences (ACEs): Leveraging the Best Available Evidence (2019), https://stacks.cdc.gov/view/cdc/82316.

[2] Q. Fan and H. Chen , “The “Long Arm” of Adverse Childhood Experiences on Adult Health Depreciation in China,” Child Abuse & Neglect 143 (2023): 106234. 37244079 10.1016/j.chiabu.2023.106234

[3] A. Danese and J. R. Baldwin , “Hidden Wounds?,” Annual Review of Psychology 68 (2017): 517–544. 10.1146/annurev-psych-010416-044208 27575032

[4] C. A. Nelson , Z. A. Bhutta , N. Burke Harris , A. Danese , and M. Samara , “Adversity in Childhood is Linked to Mental and Physical Health Throughout Life,” The BMJ 371 (2020): m3048. 33115717 10.1136/bmj.m3048 PMC7592151

[5] L. Thaler and H. Steiger , “Eating Disorders and Epigenetics,” Advances in Experimental Medicine and Biology 978 (2017): 93–103. 28523542 10.1007/978-3-319-53889-1_5

[6] A. Danese and C. S. Widom , “Objective and Subjective Experiences of Child Maltreatment and Their Relationships With Psychopathology,” Nature Human Behaviour 4, no. 8 (2020): 811–818. 10.1038/s41562-020-0880-3 32424258

[7] M. A. Reinhard , S. V. Rek , T. Nenov‐Matt , et al., “Association of Loneliness and Social Network Size in Adulthood With Childhood Maltreatment: Analyses of a Population‐Based and a Clinical Sample,” European Psychiatry 65, no. 1 (2022): e55. 36059118 10.1192/j.eurpsy.2022.2313 PMC9491078

[8] E. Kuzminskaite , A. W. Gathier , P. Cuijpers , et al., “Treatment Efficacy and Effectiveness in Adults With Major Depressive Disorder and Childhood Trauma History: A Systematic Review and Meta‐Analysis,” The Lancet Psychiatry 9, no. 11 (2022): 860–873. 36156242 10.1016/S2215-0366(22)00227-9

[9] G. Turecki , D. A. Brent , D. Gunnell , et al., “Suicide and Suicide Risk,” Nature Reviews Disease Primers 5, no. 1 (2019): 74. 10.1038/s41572-019-0121-0 31649257

[10] S. Alter , C. Wilson , S. Sun , et al., “The Association of Childhood Trauma With Sleep Disturbances and Risk of Suicide in US Veterans,” Journal of Psychiatric Research 136 (2021): 54–62. 33561736 10.1016/j.jpsychires.2021.01.030

[11] D. Lu , W. Wang , X. Qiu , et al., “The Prevalence of Confirmed Childhood Trauma and Its' impact on Psychotic‐Like Experiences in a Sample of Chinese Adolescents,” Psychiatry Research 287 (2020): 112897. 32203750 10.1016/j.psychres.2020.112897

[12] T. Zhang , L. Kan , C. Jin , and W. Shi , “Adverse Childhood Experiences and Their Impacts on Subsequent Depression and Cognitive Impairment in Chinese Adults: A Nationwide Multi‐Center Study,” Journal of Affective Disorders 323 (2023): 884–892. 36566934 10.1016/j.jad.2022.12.058

[13] J. Li , Z. Liu , M. Li , et al., “Associations of Adverse Childhood Experiences With Common Psychiatric Disorder in Later Life: Results From the China Mental Health Survey,” BMC Geriatrics 23, no. 1 (2023): 706. 37907840 10.1186/s12877-023-04421-z PMC10619228

[14] W. Xiao , S. Li , H. Xu , et al., “Population Attributable Fractions of Adverse Childhood Experiences for Emotional Problems and Self‐Harming Behaviors Among Middle School Students in China,” Asian Journal of Psychiatry 85 (2023): 103621. 37201384 10.1016/j.ajp.2023.103621

[15] D. Sun , R. Zhang , X. Ma , et al., “The Association Between Childhood Trauma and the Age of Onset in Drug‐Free Bipolar Depression,” Psychiatry Research 310 (2022): 114469. 35231875 10.1016/j.psychres.2022.114469

[16] X. Fang , D. A. Fry , and K. Ji , “The Burden of Child Maltreatment in China: A Systematic Review,” Bulletin of the World Health Organization 93, no. 3 (2015): 176–185C. 25838613 10.2471/BLT.14.140970 PMC4371492

[17] A. C. Kallstrom‐Fuqua , R. Weston , and L. L. Marshall , “Childhood and Adolescent Sexual Abuse of Community Women: Mediated Effects on Psychological Distress and Social Relationships,” Journal of Consulting and Clinical Psychology 72, no. 6 (2004): 980–992. 15612845 10.1037/0022-006X.72.6.980

[18] T. Rechenberg , T. Fleischer , C. Sander , and G. Schomerus , “Gender‐Related Stigma Toward Individuals With a History of Sexual or Physical Violence in Childhood,” BMC Public Health [Electronic Resource] 24, no. 1 (2024): 2396. 39227860 10.1186/s12889-024-19913-9 PMC11373443

[19] J. E. Lansford , L. Chang , K. A. Dodge , et al., “Physical Discipline and Children's Adjustment: Cultural Normativeness as a Moderator,” Child Development 76, no. 6 (2005): 1234–1246. 16274437 10.1111/j.1467-8624.2005.00847.x PMC2766084

[20] L. Liu and M. Wang , “Parental Harsh Discipline and Adolescent Problem Behavior in China: Perceived Normativeness as a Moderator,” Child Abuse & Neglect 86 (2018): 1–9. 30248492 10.1016/j.chiabu.2018.09.009

[21] R. Gilbert , C. S. Widom , K. Browne , D. Fergusson , E. Webb , and S. Janson , “Burden and Consequences of Child Maltreatment in High‐Income Countries,” The Lancet 373, no. 9657 (2009): 68–81. 10.1016/S0140-6736(08)61706-7 19056114

[22] M. T. Merrick , D. C. Ford , K. A. Ports , and A. S. Guinn , “Prevalence of Adverse Childhood Experiences from the 2011–2014 Behavioral Risk Factor Surveillance System in 23 States,” JAMA Pediatrics 172, no. 11 (2018): 1038–1044. 30242348 10.1001/jamapediatrics.2018.2537 PMC6248156

[23] T. W. Viola , G. A. Salum , B. Kluwe‐Schiavon , B. Sanvicente‐Vieira , M. L. Levandowski , and R. Grassi‐Oliveira , “The Influence of Geographical and Economic Factors in Estimates of Childhood Abuse and Neglect Using the Childhood Trauma Questionnaire: A Worldwide Meta‐Regression Analysis,” Child Abuse & Neglect 51 (2016): 1–11. 26704298 10.1016/j.chiabu.2015.11.019

[24] R. H. Salk , J. S. Hyde , and L. Y. Abramson , “Gender Differences in Depression in Representative National Samples: Meta‐Analyses of Diagnoses and Symptoms,” Psychological Bulletin 143, no. 8 (2017): 783–822. 28447828 10.1037/bul0000102 PMC5532074

[25] L. Gu , J. Xie , J. Long , et al., “Epidemiology of Major Depressive Disorder in Mainland China: A Systematic Review,” PLoS ONE 8, no. 6 (2013): e65356. 23785419 10.1371/journal.pone.0065356 PMC3681935

[26] R. Chen , J. An , and J. Ou , “Suicidal Behaviour Among Children and Adolescents in China,” The Lancet Child & Adolescent Health 2, no. 8 (2018): 551–553. 30119712 10.1016/S2352-4642(18)30170-6

[27] J. Zhang , Y. Liu , and L. Sun , “Psychological Strain and Suicidal Ideation: A Comparison Between Chinese and US College Students,” Psychiatry Research 255 (2017): 256–262. 28595148 10.1016/j.psychres.2017.05.046

[28] C. Bethell , J. Jones , N. Gombojav , J. Linkenbach , and R. Sege , “Positive Childhood Experiences and Adult Mental and Relational Health in a Statewide Sample,” JAMA Pediatrics 173, no. 11 (2019): e193007. 31498386 10.1001/jamapediatrics.2019.3007 PMC6735495

[29] J. Lian , K. M. Kiely , B. L. Callaghan , and K. J. Anstey , “Childhood Adversity is Associated With Anxiety and Depression in Older Adults: A Cumulative Risk and Latent Class Analysis,” Journal of Affective Disorders 354 (2024): 181–190. 38484890 10.1016/j.jad.2024.03.016

[30] L. Lin , H. H. Wang , C. Lu , W. Chen , and V. Y. Guo , “Adverse Childhood Experiences and Subsequent Chronic Diseases Among Middle‐Aged or Older Adults in China and Associations With Demographic and Socioeconomic Characteristics,” JAMA Network Open 4, no. 10 (2021): e2130143. 34694390 10.1001/jamanetworkopen.2021.30143 PMC8546496

[31] Y.‐R. Wang , J.‐W. Sun , P.‐Z. Lin , H.‐H. Zhang , G.‐X. Mu , and F.‐L. Cao , “Suicidality Among Young Adults: Unique and Cumulative Roles of 14 Different Adverse Childhood Experiences,” Child Abuse & Neglect 98 (2019): 104183. 31521907 10.1016/j.chiabu.2019.104183

[32] T. O. Afifi , M. W. Enns , B. J. Cox , G. J. G. Asmundson , M. B. Stein , and J. Sareen , “Population Attributable Fractions of Psychiatric Disorders and Suicide Ideation and Attempts Associated With Adverse Childhood Experiences,” American Journal of Public Health 98, no. 5 (2008): 946–952. 18381992 10.2105/AJPH.2007.120253 PMC2374808

[33] E. S. Howe and E. R. Dworkin , “The Day‐to‐Day Relationship Between Posttraumatic Stress Symptoms and Social Support After Sexual Assault,” European Journal of Psychotraumatology 15, no. 1 (2024): 2311478. 38376992 10.1080/20008066.2024.2311478 PMC10880566

[34] S. E. Ullman and M. Relyea , “Social Support, Coping, and Posttraumatic Stress Symptoms in Women Sexual Assault Survivors: A Longitudinal Analysis,” Journal of Traumatic Stress 29, no. 6 (2016): 500–506. 27862347 10.1002/jts.22143 PMC5140734

[35] E. N. Satinsky , B. Kakuhikire , C. Baguma , et al., “Adverse Childhood Experiences, Adult Depression, and Suicidal Ideation in Rural Uganda: A Cross‐Sectional, Population‐Based Study,” PLoS Medicine 18, no. 5 (2021): e1003642. 33979329 10.1371/journal.pmed.1003642 PMC8153443

[36] V. J. Felitti , R. F. Anda , D. Nordenberg , et al., “Relationship of Childhood Abuse and Household Dysfunction to Many of the Leading Causes of Death in Adults: The Adverse Childhood Experiences (ACE) Study,” American Journal of Preventive Medicine 14, no. 4 (1998): 245–258. 9635069 10.1016/s0749-3797(98)00017-8

[37] J. Radley , C. Grant , J. Barlow , and L. Johns , “Parenting Interventions for People With Schizophrenia or Related Serious Mental Illness,” The Cochrane Database of Systematic Reviews 10, no. 10 (2021): CD013536. 34666417 10.1002/14651858.CD013536.pub2 PMC8526162

[38] M. L. Gunlicks and M. M. Weissman , “Change in Child Psychopathology With Improvement in Parental Depression: A Systematic Review,” Journal of the American Academy of Child and Adolescent Psychiatry 47, no. 4 (2008): 379–389. 18388766 10.1097/CHI.0b013e3181640805

[39] P. Ip , R. S. Wong , S. L. Li , K. L. Chan , F. K. Ho , and C.‐B. Chow , “Mental Health Consequences of Childhood Physical Abuse in Chinese Populations: A Meta‐Analysis,” Trauma, Violence & Abuse 17, no. 5 (2016): 571–584. 10.1177/1524838015585317 25977122

[40] National Bureau of Statistics of China , Chinese Statistical Yearbook 2023 (2023), https://www.stats.gov.cn/sj/ndsj/2023/indexeh.htm.

[41] K. Kroenke , R. L. Spitzer , and J. B. Williams , “The PHQ‐9: Validity of a Brief Depression Severity Measure,” Journal of General Internal Medicine 16, no. 9 (2001): 606–613. 11556941 10.1046/j.1525-1497.2001.016009606.x PMC1495268

[42] Y. H. Lee , Z. Liu , D. Fatori , et al., “Association of Everyday Discrimination With Depressive Symptoms and Suicidal Ideation During the COVID‐19 Pandemic in the All of Us Research Program,” JAMA Psychiatry 79, no. 9 (2022): 898–906. 35895053 10.1001/jamapsychiatry.2022.1973 PMC9330278

[43] Y. Feng , W. Huang , T.‐F. Tian , et al., “The Psychometric Properties of the Quick Inventory of Depressive Symptomatology‐Self‐Report (QIDS‐SR) and the Patient Health Questionnaire‐9 (PHQ‐9) in Depressed Inpatients in China,” Psychiatry Research 243 (2016): 92–96. 27376668 10.1016/j.psychres.2016.06.021

[44] R. L. Spitzer , K. Kroenke , J. B. W. Williams , and B. Löwe , “A Brief Measure for Assessing Generalized Anxiety Disorder: The GAD‐7,” Archives of Internal Medicine 166, no. 10 (2006): 1092–1097. 16717171 10.1001/archinte.166.10.1092

[45] H. W. Fung , H. M. Chung , and C. A. Ross , “Demographic and Mental Health Correlates of Childhood Emotional Abuse and Neglect in a Hong Kong Sample,” Child Abuse & Neglect 99 (2020): 104288. 31821980 10.1016/j.chiabu.2019.104288

[46] K. Hughes , M. A. Bellis , K. A. Hardcastle , et al., “The Effect of Multiple Adverse Childhood Experiences on Health: A Systematic Review and Meta‐Analysis,” The Lancet Public Health 2, no. 8 (2017): e356–e366. 29253477 10.1016/S2468-2667(17)30118-4

